# T37. THE LONELY MOUSE: A MODEL FOR STUDYING MATERNAL PSYCHOLOGICAL STRESS AND ITS CONSEQUENCES IN THE OFFSPRING

**DOI:** 10.1093/schbul/sby016.313

**Published:** 2018-04-01

**Authors:** Joseph Scarborough, Flavia Müller, Annamaria Cattaneo, Juliet Richetto

**Affiliations:** 1 Institute for Veterinary Pharmacology and Toxicology, University of Zurich; 2 IRCCS Fatebenefratelli San Giovanni di Dio

## Abstract

**Background:**

It is well established that antenatal psychopathology affects obstetric outcomes and maternal behavior, and that it has long-term consequences on the offspring’s wellbeing and mental health, which are relevant for multiple psychiatric disorders. Against this background, it is of pivotal importance to investigate the precise mechanisms that underlie such association, and evaluate the potential beneficial and/or detrimental impact of pharmacological treatment during pregnancy and the postpartum period. To date, rodent models rely mainly on exposure to chronic or acute unpredictable stress during pregnancy, which is mainly based on physical stressors characterized by medium translational value. Therefore, we propose the use of a social isolation-rearing paradigm to investigate the effects of antenatal maternal stress on the offspring. This model has the advantage of implementing psychological stressors, as opposed to physical stressors, to induce depressive-like behaviours in female mice. Moreover, the depressive-like state can be induced and assessed before pregnancy, thus eliminating possible confounding factors that arise from physical stressful manipulations applied during pregnancy.

**Methods:**

C57BL/6 female mice were socially isolated, or group housed, from weaning (PND21) to adulthood (PND91). After 5 weeks of social isolation, the animals were tested to confirm the development of a depressive-like phenotype. At PND91, both group housed and socially isolated mice were bred and left undisturbed during pregnancy. The offspring were subjected to cognitive and behavioral testing in adulthood. A subgroup of socially isolated and grouped females were treated with the antidepressant Fluoxetine (10mg/kg) for the last 3 weeks of social isolation, pregnancy and weaning, and the offspring were once again subjected to cognitive and behavioral testing in adulthood.

**Results:**

Social isolation rearing induced weight gain, basal plasma corticosterone reduction and depressive-like behavioural traits, such as reduced social interaction and increased anxiety. Both female and male offspring of socially isolated mothers displayed a variety of behavioural abnormalities relevant to different psychiatric disorders, such as increased anxiety and altered fear expression. Male offspring also presented metabolic alterations and cognitive deficits in the form of spatial working memory and recognition memory. Prenatal fluoxetine was effective in rescuing some of the above-mentioned behavioural abnormalities but detrimental for others.

**Discussion:**

Our results demonstrate, for the first time, that long-lasting psychological stress preceding pregnancy is sufficient for inducing long-term behavioural and metabolic alterations in the offspring. Specifically, social isolation can be considered a strong etiological factor for stress in rodents, just as loneliness is a significant precursor to depression in humans. The social isolation-rearing model could thus offer a translationally-relevant setting in which to further investigate the mechanisms underlying the association between prenatal stress and psychopathology in the offspring, and the contribution of pharmacological treatments.

